# Role of Combined Lipoic Acid and Vitamin D3 on Astrocytes as a Way to Prevent Brain Ageing by Induced Oxidative Stress and Iron Accumulation

**DOI:** 10.1155/2019/2843121

**Published:** 2019-02-28

**Authors:** Claudio Molinari, Vera Morsanuto, Sabrina Ghirlanda, Sara Ruga, Felice Notte, Laura Gaetano, Francesca Uberti

**Affiliations:** ^1^Laboratory of Physiology, Department of Translational Medicine, University of Piemonte Orientale, via Solaroli 17, Novara 28100, Italy; ^2^Contract Research Organization (MIAC AG) Affiliated to the University Hospital of Basel, Mittlere Strasse 83 CH-4031 Basel, Switzerland

## Abstract

Brain ageing is a complex multifactorial process characterized by gradual and continuous loss of neuronal functions. It is hypothesized that at the basis of brain ageing as well as age-related diseases, there is an impairment of the antioxidant defense system leading to an increase of oxidative stress. In this study, two different biological aspects involved in brain ageing and neurodegeneration have been investigated: oxidative stress and iron accumulation damage. In primary mouse astrocytes, the stimulation with 50 *μ*M lipoic acid (LA) and 100 nM vitamin D (vitD) was first investigated in a time-course study to determine the dosages to be used in combination and then in a permeability test using an *in vitro* blood-brain barrier. In a second set of experiments, the role of oxidative stress was investigated pretreating astrocytes with 200 *μ*M H_2_O_2_ for 30 min. The ability of vitD and LA alone and combined together to prevent or repair the damage caused by oxidative stress was investigated after 24 h of stimulation by the MTT test, mitochondrial membrane potential measurement, and Western blot analysis. To induce neurodegeneration, cells were pretreated with 300 *μ*M catalytic iron for 6 days and then treated with vitD and LA alone and combined for additional 6 days to investigate the protection exerted by combination, analyzing viability, ROS production, iron concentration, and activation of intracellular pathways. In our study, the combination of LA and vitD showed beneficial effects on viability of astrocytes, since the substances are able to cross the brain barrier. In addition, combined LA and vitD attenuated the H_2_O_2_-induced apoptosis through the mitochondrial-mediated pathway. The combination was also able to counteract the adverse conditions caused by iron, preventing its accumulation. All these data support the hypothesis of the synergistic and cooperative activity exerted by LA and vitD in astrocytes indicating a possible new strategy to slow down ageing.

## 1. Introduction

Ageing is characterized by a progressive loss of physiological integrity, leading to impaired function of organs and systems and increased vulnerability to death. This deterioration is the primary risk factor for major human pathologies including cancer, diabetes, cardiovascular disorders, and neurodegenerative diseases [1]. Ageing is a multifactorial process, which includes genomic instability, telomere attrition, epigenetic alterations, loss of protein homeostasis, deregulated nutrient sensing, mitochondrial dysfunction, cellular senescence, stem cell exhaustion, and altered intercellular communication [1]. The effect of ageing on the brain, in particular on molecules, cells, vascularization, gross morphology, and cognition, is widespread and has multiple etiologies [2]. It is widely recognized that biological ageing is not closely linked to chronological ageing, and the scientific community is trying to make it possible to slow down biological ageing and to reduce the possibility of suffering from age-related diseases such as the neurodegenerative ones. Cognitive frailty is emerging as one of the greatest health threats of the twenty-first century. Life expectancy continues to grow, but in the same time, the prevalence of cognitive decline is increasing [3]. This is becoming a serious social problem that causes discomfort and increase of costs for individuals, families, and health systems [4]. Neurodegenerative diseases are a heterogeneous group of disorders characterized by progressive and selective neuronal death leading to the degeneration of specific brain regions predominantly caused by the natural ageing process [5]. The most common neurodegenerative diseases are Alzheimer's disease (AD) characterized by progressive loss of cognitive functions [6] and Parkinson's disease (PD) characterized by motor symptoms related to dopaminergic neuronal loss in the substantia nigra [7]. The molecular, cellular, anatomical, and neurochemical changes in the brain are all associated with ageing and age-associated diseases [8]. A healthy ageing brain is characterized by decline in neuronal activity, loss of synaptic connection and function [9]. In this context, main important functions are exerted by astrocytes, members of nonneuronal glial cells, which regulate synaptic formation and synaptic communication and support neuronal health and homeostasis [10].

During ageing, both cortical volume and neuronal activity worsen, and this leads to the atrophy of neuronal dendrites and to the reduction of the dendritic spine number [11]. The most important functions of astrocytes include the recycling of neurotransmitters, support for facilitating neuronal transmission [12], and the transport of nutrients such as lipids and sugars to produce energy for neurons [13]. The functions of astrocytes, so important for the central nervous system, appear altered in the ageing brain, and this causes the reduction of neuronal and synaptic functions. Oxidative stress plays a key role in astrocyte loss; in particular, it is due to a highly active mitochondria metabolism [14].

The mitochondrial “free radical” theory is one of the most studied hypotheses to explain the molecular mechanism of ageing; it is based on the endogenous production of reactive oxygen species (ROS) and their harmful effects on the mitochondria [15]. Moreover, nitric oxide (NO) when produced in high concentrations works as a source of toxic oxidants, called reactive nitrogen species [16]. ROS react with lipids, proteins, and nucleic acids, causing oxidative damage, leading to progressive decline of cellular functions [17]. Peroxidation of membrane lipids has been hypothesized to be a major mechanism of ROS attack, resulting in generalized impairment of the membrane function [18]. On the other hand, reactive nitrogen species have been involved in diseases that are related to neurotransmission and cancer where NO is produced at high concentrations from nonvascular cell sources. Conversely, NO and drugs that directly or indirectly increase NO signaling have found clinical applications in both age-related diseases and in younger individuals [19]. Anyway, the organism possesses defense mechanisms based on antioxidant actions; a group of enzymatic (e.g., superoxide dismutase, catalase, and glutathione reductase) or nonenzymatic (e.g., glutathione, melatonin, vitamins A, C, and E, and flavonoids) molecules that plays an important role in maintaining homeostasis and cell viability [14]. In some cases, the endogenous antioxidant system is not strong enough to counteract the oxidative damage caused by ROS and NO. For this reason, several studies have been conducted to investigate the effects of antioxidant dietary supplementation in ageing or neurodegenerative diseases [20, 21]. Indeed, an increased consumption of specific nutrients such as polyphenols, fish and seafood, vitamins, and oligoelements demonstrated protective effects by targeting specific cellular markers and functions [8].

In scientific literature, a number of clinical data regarding the effects of supplemental substances (i.e., polyphenols, vitamins, oligoelements, and *ω*-3 polyunsaturated fatty acids) are available, demonstrating encouraging therapeutic properties on neurodegenerative processes related to brain ageing [8]. Nutraceutical interventions can slow down physiological or/and pathological progression due to their antioxidant, anti-inflammatory, and antiamyloidogenic properties, regulating mitochondrial stress, apoptotic factors, free radical scavenging system, and neurotrophic factors [8, 22]. Finally, a mechanism typically present in healthy ageing is the selective accumulation of iron that occurs in several brain regions and cell types, with iron mainly bound within ferritin and neuromelanin [23]. However, the accumulation of iron in specific brain regions, greater than that reported in healthy ageing, occurs in many neurodegenerative diseases and is often associated with oxidative stress and cellular damage [24]. Whether iron accumulation found in neurodegenerative diseases is a primary event or a secondary effect is unclear. Ageing is the major risk factor for neurodegeneration. Age-related iron accumulation might be an important factor that contributes to neurodegenerative processes [24]. In this context, the use of *α*-lipoic acid (LA) and vitamin D3, which are well known to exert beneficial effects on the brain, can be hypothesized. Indeed, both LA and vitamin D3 are able to cross the blood-brain barrier (BBB) [25, 26], and several studies have been performed to understand their functions within the ageing process of the brain [5, 27–29].

Vitamin D or calciferol is a fat-soluble secosteroid synthesized in skin or ingested with food which has only one active form named vitamin D3 (vitD), cholecalciferol, or 1*α*,25(OH)_2_D_3_ [30]. VitD regulates the expression of numerous target genes through the nuclear vitamin D receptor (VDR), which controls downstream events including the protective role against oxidative stress, regulation of autophagic pathways [31], and interplay between apoptosis and survival pathways [32]. LA (1,2-dithiolane- 3-pentanoic acid) is a naturally occurring dithiol compound synthesized enzymatically in the mitochondrion from octanoic acid. LA's antioxidant properties include its ability to directly scavenge ROS, to regenerate endogenous antioxidants, such as glutathione and vitamins E and C, and to have a metal-chelating activity [33]. Even on its reduced form, the dihydrolipoic acid (DHLA) is considered an antioxidant compound [34]. In addition, brain ageing has been also related to inflammation [35], induced by ROS that modulates cellular mechanisms for cell proliferation and survival, death, and immune responses by inducing the production of proinflammatory factors such as cytokines leading to cognitive dysfunctions and memory loss [36]. Furthermore, excessive secretion of proinflammatory cytokines has been established to be a crucial promoter of aberrant inflammatory responses in neurodegenerative diseases [37]. LA is considered as a potential anti-inflammatory agent for neurodegenerative conditions such as Alzheimer's disease, showing neuroprotective effects in various neuronal experimental models, and a modulator of various genes involved in cell survival, inflammation, and oxidative stress [38]. The aim of this study was to test the efficacy of vitD to prevent the loss of astrocytes during oxidative stress and iron accumulation. In addition, the ability of LA to alleviate the effects of oxidative stress, enhance the endogenous antioxidant action, and chelate iron preventing its accumulation in the cells was assessed. Moreover, it was evaluated whether there is a cooperation between LA and vitD in order to hypothesize a new human antiageing treatment, such as a new food supplement called Cebral®.

## 2. Materials and Methods

### 2.1. Astrocyte Isolation and Culture

Primary mouse astrocyte cultures were prepared from both male and female C57BL/6 mouse pups, following a classical technique described elsewhere [39] according to the National Guideline for the Use and Care of Laboratory Animals. Briefly, within 24 h of birth, pups were euthanized, and cortices were dissected, minced, mechanically digested, and let settle for 30 min at room temperature. Then the cell suspension was centrifuged at 800 rpm for 5 min. Pelleted cells were resuspended in Neuronal Basal Medium (Sigma-Aldrich), supplemented with 5% fetal bovine serum (FBS, Gibco), 1% penicillin/streptomycin (Sigma-Aldrich), and 2 mM L-glutamine (Sigma-Aldrich), plated in multiwells, and maintained in culture for 6 days before treatment. Astrocytes should be separated from microglia and oligodendrocyte precursor cells by shaking, as reported in literature [40]. For the experiments, 1 × 10^4^ cells on a 96-well plate were plated to study cell viability by the MTT test, amyloid precursor protein (APP) by the ELISA test, and ROS production by the colorimetric test; 1 × 10^4^ cells on a black 96-well plate to analyze the oxygen consumption by a fluorescence kit; 1 × 10^6^ on a 6-well plate to determine the iron concentration by the colorimetric assay; 1 × 10^6^ on a 6-well plate to analyze the intracellular pathways activated by Western blot analysis; 1 × 10^6^ on a 6-well plate to analyze p53 activity and ERK/Akt activation; and 4 × 10^4^ on Transwell support to study the permeability, to quantify vitD and LA. Before stimulations, the cells were maintained in Dulbecco's modified Eagle's medium (DMEM, Sigma-Aldrich) without red phenol and fetal bovine serum (FBS, Sigma-Aldrich) and supplemented with 1% penicillin/streptomycin (Sigma-Aldrich) and 2 mM L-glutamine (Sigma-Aldrich) in an incubator at 37°C, 5% CO_2_, and 95% humidity for 3 h.

### 2.2. HUVEC Culture

In this study, human umbilical endothelial cells (HUVEC) were also used, in order to obtain a coculture together with astrocytes as an experimental *in vitro* model of BBB. HUVEC were purchased from ATCC®. Cells were cultured in EGM Media (Lonza) supplemented with 10% FBS (Gibco), 1% penicillin/streptomycin (Sigma-Aldrich), and 2 mM glutamine (Sigma-Aldrich) at 37°C in a humidified atmosphere of 95% air, 5% CO_2_. For the experiments, 1 × 10^5^ HUVEC cells/cm^2^ were plated in the apical compartment of 6.5 mm Transwells with a 0.4 *μ*m pore size polyester membrane (Corning Costar, Sigma).

### 2.3. Experimental Protocol

The cells were used to study two different biological aspects involved in brain ageing and neurodegeneration: oxidative stress and iron-dependent damage. In the first set of experiments, the role of vitD and LA under physiological condition was analyzed. In this step, the dose- and time-dependent studies (from 15 min to 1440 min) on cell viability were performed with LA (from 10 *μ*M to 100 *μ*M) to determine its optimal concentration, and then this concentration (50 *μ*M) was maintained in all successive experiments. Then the combination with 50 *μ*M LA and 100 nM vitD [41, 42] was investigated in a time-course study (from 15 min to 1440 min) and then by the permeability assay to determine each specific concentration through BBB. In a second set of experiments, the role of oxidative stress was investigated by pretreatment for 30 min with 200 *μ*M H_2_O_2_ on astrocytes [43]. Particularly, the ability of vitD and LA alone and combined to prevent or restore the damage caused by oxidative stress was analyzed by the MTT test. Moreover, mitochondrial membrane potential measurement, amyloid precursor protein (APP) quantification, and Western blot analysis were performed at 24 h. In a third set of experiments, to induce neurodegeneration, cells were pretreated with catalytic iron (Fe^3+^) 300 *μ*M for 6 days [44] and then treated with vitD and LA alone and combined for additional 6 days to investigate the protection exerted by the combination, analyzing viability, ROS production, iron concentration, APP quantification, and activated intracellular pathways.

### 2.4. MTT Test

MTT-based In Vitro Toxicology Assay Kit (Sigma-Aldrich) was performed on a 96-well plate to determine cell viability after each stimulation as previously described [45]. At the end of stimulation, cells were incubated with 1% MTT dye for 2 h at 37°C in an incubator, 5% CO_2_, and 95% humidity, and then, the purple formazan crystals were dissolved in equal volume of MTT Solubilization Solution. Cell viability was determined by measuring the absorbance at 570 nm with correction at 690 nm, through a spectrometer (VICTOR X4, multilabel plate reader), and calculated by comparing results to the control.

### 2.5. Blood-Brain Barrier Experimental Model

Astrocytes were cocultured with HUVEC cells according to methods reported in literature [46]. In brief, 4 × 10^4^ astrocytes/cm^2^ were plated on the basolateral side of the flipped 6.5 mm Transwells with a polyester membrane with 0.4 *μ*m pore size (Corning Costar, Sigma-Aldrich) and left to attach for 4 h. Transwells were then placed into the normal orientation and the cells left to grow for 48 h. After this time, 1 × 10^5^ HUVEC cells/cm^2^ were plated in the apical compartment. The inserts were then placed in a 24-well plate. After 7 days of culture, the Transwells were treated, and permeability studies were performed [47]. To understand the ability of tested substances to cross the blood-brain barrier, the medium at the bottom side of the Transwells was quantified over time (from 15 min to 1440 min) by measuring the volume and the concentration of vitD and LA.

### 2.6. Lipoic Acid Determination

The concentration of LA which crossed to BBB was measured as described in literature [48]. Briefly, at the end of stimulations, the basolateral volume was analyzed through a spectrometer (VICTOR X4, multilabel plate reader) at 320 nm, and the absorbance related to the standard curve was obtained from LA (200 ng/ml). The results were expressed as means ± SD (%) of absorption, normalized to the control.

### 2.7. Vitamin D Quantification

The competitive ELISA assay kit (FineTest) has been used to detect the metabolically active form of vitD, such as 25(OH)D3. At the end of each stimulation, 50 *μ*l of each sample was collected and immediately used, following the manufacturer's instruction. At each sample, 50 *μ*l of biotin-detection and 100 *μ*l of SABC working solution were added and incubated at 37°C for 30 min. At the end, the supernatants were discarded, and then 90 *μ*l TMB substrate plus and 50 *μ*l of stop solution were added. Finally, the 96-well plate was analyzed by a spectrometer at 450 nm (VICTOR X4, multilabel plate reader). In addition, it was necessary to plot a standard curve including the background (zero well) to perform a quantification.

### 2.8. ROS Production

The rate of superoxide anion release was used to examine ROS produced by astrocytes after stimulations [41]. After treatment, in all samples (treated or not), 100 *μ*l of cytochrome C was added, and in another sample, 100 *μ*l superoxide dismutase was also added for 30 min in an incubator (all substances were from Sigma-Aldrich). The absorbance was measured by a spectrometer (VICTOR X4, multilabel plate reader), at 550 nm, and O_2_ was expressed as means ± SD of nanomoles per reduced cytochrome C per microgram of protein compared to the control on percentage (%).

### 2.9. Mitochondrial Membrane Potential

The mitochondrial membrane potential was analyzed by the Oxygen Consumption/Mito membrane Potential Dual Assay Kit (Cayman Chemical Company) following manufacturer's instructions [49]. The mitochondrial membrane potential was measured using JC-1 aggregates at an excitation/emission of 560/590 nm and monomers at an excitation/emission of 485/535 nm in a fluorescence spectrometer (VICTOR X4, multilabel plate reader). The results are expressed as means ± SD (%) compared to control cells.

### 2.10. p53 Activity

p53 activity was measured by the specific ELISA kit (p53 transcription factor assay kit, Cayman Chemical), examining the nuclear extracts obtained at the end of each stimulation following the manufacturer's instructions. The nuclear extraction was obtained by the classical technique using a complete buffer present in the kit. Briefly, the cells were lysed with ice-cold 1X Complete Hypotonic Buffer, supplemented with NP-40, and then centrifuged at 12,000 g at 4°C for 10 min. The pellet was solubilized with ice-cold Complete Nuclear Extraction Buffer 1x supplemented with protease and phosphatase inhibitors and then centrifuged at 12,000 g for 15 min at 4°C; the supernatant was examined to analyze the activity of p53 related to the protein quantification through the BCA assay (Thermo Fisher).

### 2.11. Amyloid Precursor Protein (APP) Quantification

Amyloid precursor protein (APP) quantification was measured by the Amyloid Beta A4 protein ELISA kit (Sigma-Aldrich) on cellular supernatants following the manufacturer's instructions. Briefly, at the end of treatments, cellular supernatants were collected, and each sample was tested with the ELISA kit. The biotinylated detection antibody specific for the target protein was added in each well, and the plate was incubated for 1 hour at room temperature. Then, after 45 minutes of incubation with HRP-conjugated streptavidin, TMB substrate solution was added for 30 minutes, and subsequently, the reaction was stopped by adding stop solution. APP concentration was determined by measuring the absorbance through a spectrometer (VICTOR X4, multilabel plate reader) at 450 nm and calculated by comparing results to the APP standard curve.

### 2.12. ERK and Akt Activation Assay

ERK/MAPK and PI3K/Akt activities were measured by the InstantOne™ ELISA (Thermo Fisher) on cell lysates following the manufacturer's instructions [50]. Briefly, cells at the end of treatments were lysed with 100 *μ*l Cell Lysis Buffer Mix, 50 *μ*l/well of each sample was tested in InstantOne ELISA microplate strips, and at each well, the Antibody Cocktail was added and incubated for 1 h at room temperature on a microplate shaker. At the end, the Detection Reagent was added to each well, and after 20 min, the reaction was stopped by adding stop solution to each. The strips were measured by a spectrometer (VICTOR X4, multilabel plate reader) at 450 nm. The results were expressed as means absorbance (%) compared to the control.

### 2.13. Iron Quantification Assay

Iron Assay Kit (Sigma-Aldrich) which measures ferrous iron (Fe^2+^), ferric iron (Fe^3+^), and total iron (total iron–ferrous iron) in samples was used on astrocytes following the manufacturer's instructions [45]. The absorbance at 593 nm was measured by a spectrometer (VICTOR X4, multilabel plate reader). Ferric iron concentrations are equal to the total iron (sample plus iron reducer)-Fe^2+^ (sample plus assay buffer). The iron concentration was expressed as ng/ml.

### 2.14. Western Blot

Cells were washed and then lysed in the ice Complete Tablet buffer (Roche) supplemented with 2 mM sodium orthovanadate, 1 mM phenylmethanesulfonyl fluoride (PMSF; Sigma-Aldrich), and 1 : 100 mix Protease Inhibitor Cocktail (Sigma-Aldrich). From each lysate, 35 *μ*g proteins was resolved into 8% and 15% SDS-PAGE gels, and polyvinylidene difluoride (PVDF) membranes (GE Healthcare) were incubated overnight at 4°C with a specific primary antibody: anti-SOD3 (1 : 250, Santa Cruz), anti-phospho-PKA*α*/*β*/*γ* (Thr198, 1 : 250, Santa Cruz), anti-NOS2 (1 : 250, Santa Cruz), and anti-cytochrome C (1 : 1000, Calbiochem). Protein expression was normalized and verified through *β*-actin detection (1 : 5000; Sigma-Aldrich) and expressed as a mean ± SD (% vs. control).

### 2.15. Statistical Analysis

For each experimental protocol, at least four independent experiments have been carried out; the results are expressed as means ± SD of independent experiments performed on four technical replicates. One-way ANOVA followed by the Bonferroni post hoc test was used for statistical analysis, and pairwise differences compared by Mann–Whitney *U* tests followed by Welch's test. *p* values <0.05 were considered statistically significant.

## 3. Results

### 3.1. Cell Viability under Treatments with VitD and LA during Time

**Figure 1:**
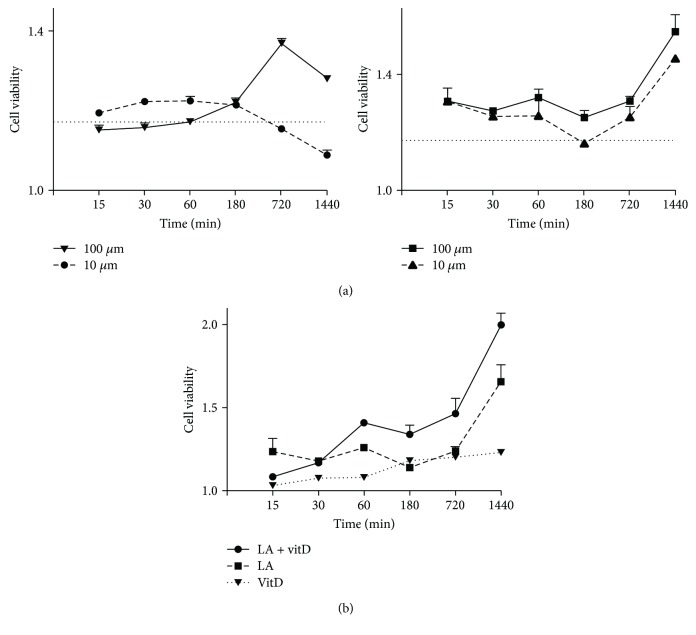
Cell viability measured in time-course and dose-response studies in astrocytes. In (a), time-course and dose-response (ranging from 10 *μ*M to 100 *μ*M) studies of LA measured in astrocytes are illustrated; in (b), time-course and dose-response studies of 50 *μ*M LA combined with 100 nM vitD measured in astrocytes are reported. LA = lipoic acid, vitD = vitamin D3, and LA+vitD = combination of lipoic acid and vitamin D3. Data are expressed as means ± SD (%) of five independent experiments normalized to the control (number 1 in the *y* axis corresponds to 100% control values).

In order to assess the potential effect of vitD and LA alone and combined on cell viability of astrocytes, the MTT test was performed both in a dose-response and in a time-course study. Firstly, the concentration-dependent effect of LA alone (ranging from 10 *μ*M to 100 *μ*M) on cell viability during 24 hours (starting from 15 min to 1440 min) was analyzed in order to identify the best concentration to use. As shown in Figure 1(a), 50 *μ*M LA appeared to be the dose able to induce the greatest effect (*p* < 0.05) compared to the control and to other concentrations (10, 25, and 100 *μ*M, *p* < 0.05) during all the time of stimulation, and the maximum effect of about 66% compared to the control was observed at 1440 min. This concentration of LA was maintained for all successive experiments. Since the new hypothesized formulation includes LA and vitD, additional experiments were carried out to study the combination of 50 *μ*M LA and 100 nM vitD on cell viability. As reported in Figure 1(b), vitD increased cell viability of astrocytes in a time-course manner with a maximum effect, about 22% (*p* < 0.05) at 1440 min compared to the control. In addition, the combination of LA and vitD was able to significantly increase (*p* < 0.05) cell viability during time compared to the control (*p* < 0.05) and to 50 *μ*M LA and 100 nM vitD alone starting from 60 min of stimulation (*p* < 0.05). The combination exerted a greater effect at 1440 min compared to the control (*p* < 0.05) and to 50 *μ*M LA and 100 nM vitD alone (about 20.5% and 64%, respectively). For this reason, this time of stimulation was maintained for all successive experiments. These data support the hypothesis of the synergistic effects exerted by vitD and LA in astrocytes indicating a possible new strategy to slow down ageing.

### 3.2. Permeability of VitD and LA through Blood-Brain Barrier (BBB)

**Figure 2:**
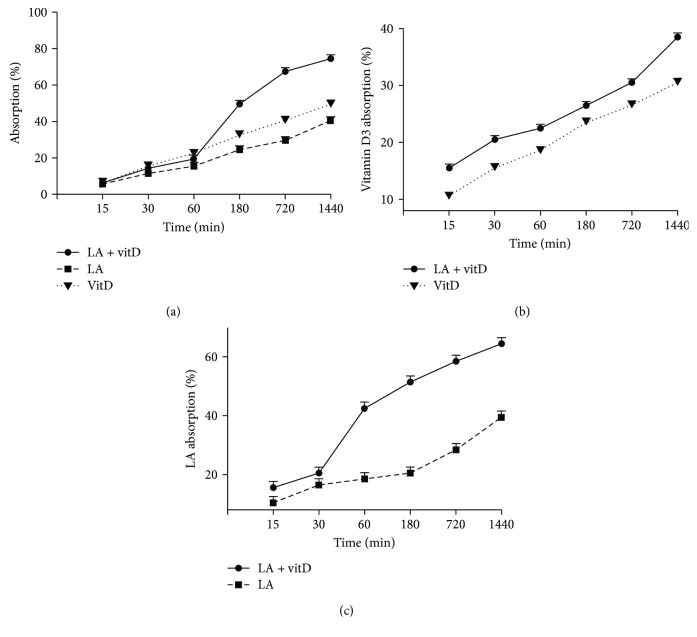
BBB permeability, vitD, and LA quantifications to predict their bioavailability in the brain. In (a), the absorption capacity through the BBB of vitD and LA alone and combined is shown; in (b), quantification of vitD is shown; and in (c), quantification of LA at basolateral environment of the barrier model are reported. The abbreviations are the same as used in Figure 1. Data are expressed as means ± SD (%) of five independent experiments normalized to control values (0% line).

A BBB permeability study was performed to better understand the ability of 100 nM vitD and 50 *μ*M LA alone and combined together during time to cross the haematoencephalic barrier. As reported in Figure 2(a), the analysis of the basolateral volume showed a time-dependent increase on absorption capacity caused by 100 nM vitD and 50 *μ*M LA alone compared to the control (*p* < 0.05), and the greater effects were observed at 1440 min (about 49.5% and 40.5%, respectively). The combination of vitD and LA increased the absorption capacity with respect to the control (*p* < 0.05) during time and to their single administration starting from 60 min, as previously observed on cell viability (*p* < 0.05). These data support a cooperative effect of vitD and LA also during the permeability assay. The successive quantifications of vitD and LA were carried out to determine the specific concentration present in basolateral volume of the BBB model. In particular, the absorption of vitD and LA during time was time-dependent, and the combination of vitD and LA proved to be essential to amplify their ability to cross the barrier. Indeed, the specific quantifications of vitD (Figure 2(b)) and LA (Figure 2(c)) showed a greater effect of the combination compared to the separated administration (about 26% and 63%, respectively), with a maximum effect at 1440 min (*p* < 0.05 vs. control). All these findings support the hypothesis that the combination of LA and vitD is able to exert beneficial effects directly on viability of astrocytes due to their ability to cross the BBB.

### 3.3. Analysis of Mitochondrial Activity after Treatments with LA and VitD under Oxidative Condition

**Figure 3:**
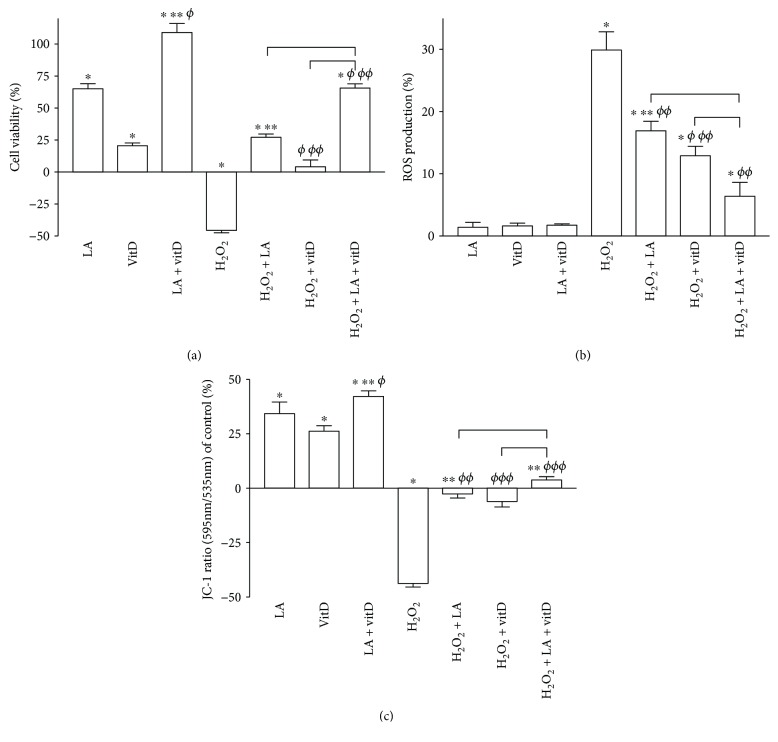
Analysis of cellular viability, ROS production, and mitochondrial activity under oxidative condition. In (a), cell viability, in (b), ROS production, and in (c), mitochondrial membrane potential measured on astrocytes treated with vitD and LA alone and combined for 24 h prestimulation with H_2_O_2_ are illustrated. The abbreviations are the same as reported in Figure 1. Data are expressed as means ± SD (%) of five independent experiments normalized to control values (0% line). ^∗^*p* < 0.05 vs. control; ^∗∗^*p* < 0.05 vs. LA; ^*ϕ*^*p* < 0.05 vs. vitD; ^*ϕϕ*^*p* < 0.05 vs. H_2_O_2_, bars: *p* < 0.05 between treatments.

Cell viability, ROS production, and mitochondrial potential were evaluated in astrocytes, in order to investigate the potential action to prevent cellular ageing under oxidative condition. Exposure to 200 *μ*M H_2_O_2_ significantly reduced cell viability of about 46% compared to the control; conversely, following posttreatment with 50 *μ*M LA and 100 nM vitD alone or combined, the cell viability was significantly increased. The greatest effect was obtained with the combination of LA and vitD which reverted the cell loss (Figure 3(a)). Since the main theory at the basis of brain ageing regards the oxidative condition, additional experiments on ROS production were performed. 50 *μ*M LA, 100 nM vitD, and the combination of both were able to maintain the ROS production under the physiological level (*p* > 0.05 vs. control), supporting the hypothesis of their safety during use (Figure 3(b)). Exposure of astrocytes to 200 *μ*M H_2_O_2_ significantly increased the intracellular ROS production as illustrated in Figure 3(b) of about 30% compared to the control (*p* < 0.05); posttreatment with 50 *μ*M LA and 100 nM vitD alone significantly reduced ROS production (about 43% and 57%, respectively, vs. H_2_O_2_ alone), and the concomitant administration of LA and vitD improved the reduction of the ROS level compared to 200 *μ*M H_2_O_2_, 50 *μ*M LA, and 100 nM vitD alone (*p* < 0.05, about 78%, 62%, and 50%, respectively). Since the alteration of the formation of a proton gradient across the inner mitochondrial membrane is considered to be one of the key indicators of cellular viability, the mitochondrial potential was analyzed. Treatments with 50 *μ*M LA, 100 nM vitD, and with the combination of both induced a significant increase in red fluorescence, supporting the active role of 50 *μ*M LA, 100 nM vitD, and their combination on mitochondrial activity (*p* < 0.05). In addition, the combination of LA and vitD seems to have a greater effect compared to 50 *μ*M LA and 100 nM vitD alone (about 23% and 60%, respectively). H_2_O_2_-treated cells exhibited changes in the fluorescence signal which lead to a decreased red fluorescence signal and increased green fluorescence signal, indicating a significant dissipation of mitochondrial potential and cell loss compared to the control (*p* < 0.05, Figure 3(c)). Posttreatment with 50 *μ*M LA, 100 nM vitD alone, and with the combination of both significantly reversed dissipation of mitochondrial potential as shown in Figure 3(c) compared to 200 *μ*M H_2_O_2_ alone (*p* < 0.05). In particular, the combination of LA and vitD suppressed the effect of H_2_O_2_-induced mitochondrial dissipation, shifting the fluorescence signal from green to red (*p* < 0.05). These results indicate that the combination of LA and vitD attenuates the H_2_O_2_-induced apoptosis through the mitochondrial-mediated pathway.

### 3.4. Study of Intracellular Pathways Activated by LA and VitD under Oxidative Condition

**Figure 4:**
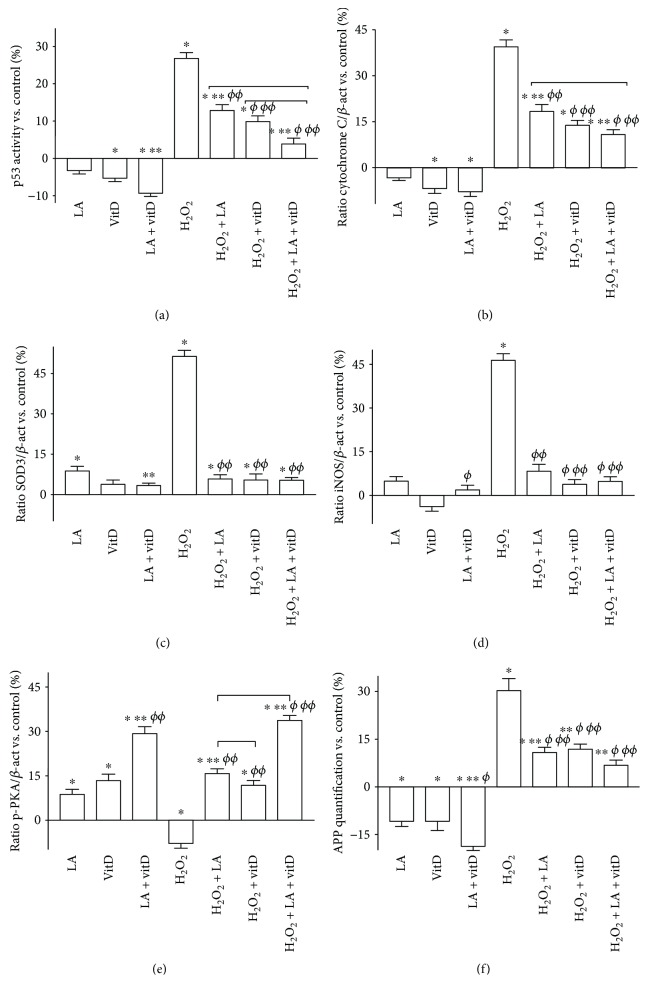
Kinase activity and densitometric analysis of intracellular pathways involved under oxidative stress. (a) and (f) The measurements of p53 and APP activities, respectively, measured by the ELISA test. (b–e) Densitometric analysis of cytochrome C, SOD3, iNOS, and p-PKA expressions obtained by analyzing Western blot on whole astrocyte lysates. The abbreviations are the same as reported in Figure 1. Data are expressed as means ± SD (%) of five independent experiments normalized to control values (0% line) in ELISA experiments and normalized and verified on *β*-actin detection in densitometric analysis expressed as means ± SD (%). ^∗^*p* < 0.05 vs. control; ^∗∗^*p* < 0.05 vs. LA; ^*ϕ*^*p* < 0.05 vs. vitD; ^*ϕϕ*^*p* < 0.05 vs. H_2_O_2_, bars: *p* < 0.05 between treatments.

**Figure 5:**
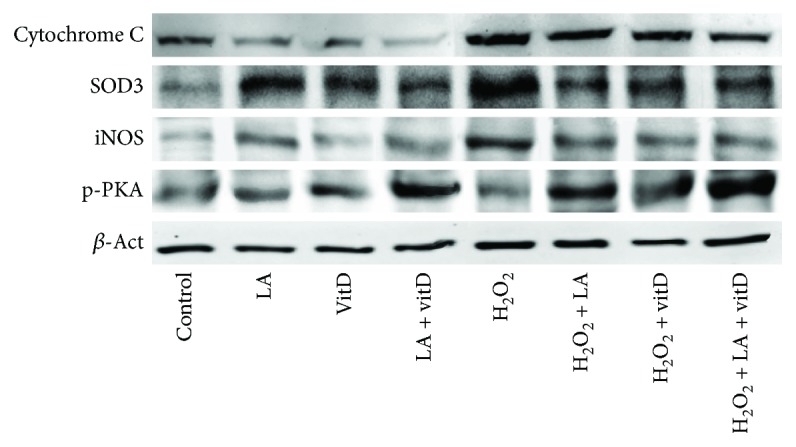
Western blot of SOD3, iNOS, and p-PKA in astrocytes under oxidative stress. The images reported are an example of each protein of five independent experiments reproduced in triplicates. The abbreviations are the same as reported in Figure 1.

The dissipation of mitochondrial potential under oxidative condition is known to initiate a cascade of events leading to the activation of caspases, which in turn trigger apoptosis. In this context, p53 as a key factor involved in ageing, oxidative stress, and neurodegeneration and cytochrome C as a key regulator of both cellular energetic metabolism and apoptosis were investigated in astrocytes. Data reported in Figure 4(a) showed a reduction on p53 activity after the stimulation with 50 *μ*M LA and 100 nM vitD alone and in combination (*p* < 0.05 vs. control), supporting previous data about the safety of the combination. p53 activity significantly increased in H_2_O_2_-treated astrocytes (*p* < 0.05 vs. control), and the subsequent stimulation with 50 *μ*M LA and 100 nM vitD alone significantly reduced it (*p* < 0.05). In addition, the combination of LA and vitD amplified the reduction compared to 200 *μ*M H_2_O_2_ of about 80% and the single administration of about 69% and 60%, respectively (*p* < 0.05), resulting in survival-favored conditions. To examine the involvement of the caspase pathways, the expression of cytochrome C was analyzed under the same conditions. Figures 4(b) and 5 show similar results for p53 under the stimulation with 50 *μ*M LA and 100 nM vitD alone, confirming the previously observed beneficial effects. The combination of LA and vitD also maintained cytochrome C at the basal level, supporting mitochondrial integrity. In addition, H_2_O_2_-treated cells showed an increase in the expression of cytochrome C which was reduced by the following stimulation with both 50 *μ*M LA and 100 nM vitD alone and combined (*p* < 0.05). In order to exclude any oxidative damage induced by the stimulations, SOD3 and iNOS expressions were investigated. As reported in Figures 4(c), 4(d), and 5, the SOD3 and iNOS expressions significantly increased in the presence of 200 *μ*M H_2_O_2_ (*p* < 0.05 vs. control), supporting the hypothesis of the involvement of oxidative stress in astrocyte death. In addition, the poststimulation with 50 *μ*M LA and 100 nM vitD alone significantly reduced the expression of both SOD3 and iNOS compared to 200 *μ*M H_2_O_2_ alone, and a greater reduction was obtained by the combined stimulation with 50 *μ*M LA and 100 nM vitD compared to 200 *μ*M H_2_O_2_ (*p* < 0.05), indicating a beneficial effect in counteracting the ageing process. Since the neuroinflammation is a common cause of brain ageing, PKA, a key anti-inflammatory marker, was also investigated under the same conditions (Figures 4(e) and 5). The expression of PKA observed in astrocytes showed a significant increase in the presence of both 50 *μ*M LA and 100 nM vitD alone (*p* < 0.05), and their combination amplified this effect supporting the anti-inflammatory activity of LA and vitD. In the presence of 200 *μ*M H_2_O_2_, a significant reduction compared to the control was found, and the poststimulation with 50 *μ*M LA and 100 nM vitD reverted the mechanism (*p* < 0.05). The combination of LA and vitD added after 200 *μ*M H_2_O_2_ had a similar effect on PKA expression to what was observed without H_2_O_2_ indicating the ability of the combination to prevent the induction of inflammatory cascade under oxidative stress. A natural consequence of apoptosis is known to be cell loss, and the *β*-amyloid analysis demonstrated the alteration in brain tissue. As reported in Figure 4(f), the stimulation with 200 *μ*M H_2_O_2_ alone caused a significant increase of the APP level, supporting previous data about cell death. In addition, the poststimulations with 50 *μ*M LA and 100 nM vitD were able to reduce the damage as shown by the decrease in the APP level (*p* < 0.05) compared to 200 *μ*M H_2_O_2_ alone. Finally, the greater effect was observed in the presence of the combined treatment with LA and vitD, indicating the effectiveness of the combination during brain damage.

**Figure 6:**
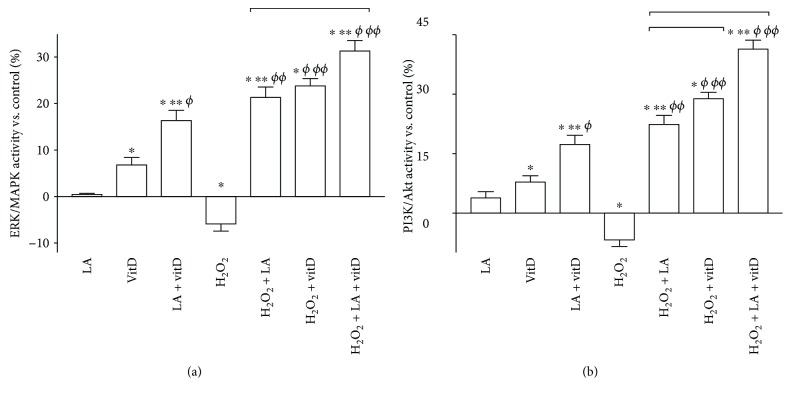
Survival kinase activities measured in astrocytes under oxidative conditions. The quantification of p-ERK (a) and p-Akt (b) activities measured at 24 h in astrocytes treated with vitD and LA alone and combined under prestimulation with H_2_O_2_ is reported. Data are reported as means ± SD (%) of five biological replicates normalized to control values (0% line). ^∗^*p* < 0.05 vs. control; ^∗∗^*p* < 0.05 vs. LA; ^*ϕ*^*p* < 0.05 vs. vitD; ^*ϕϕ*^*p* < 0.05 vs. H_2_O_2_, bars: *p* < 0.05 between treatments.

The ERK/MAPK and PI3K-Akt pathways play a crucial role in the regulation of neuronal and brain survival. 50 *μ*M LA and 100 nM vitD alone confirmed their ability to improve the viability of cells, activating ERK and Akt mediators, as reported in Figures 6(a) and 6(b). The combination of LA with vitD amplified kinase activation compared to the control and to single administration as well (*p* < 0.05). Exposure to 200 *μ*M H_2_O_2_ significantly reduced ERK and Akt activities of about 6% and 7%, respectively, compared to the control; conversely, subsequent posttreatment with 50 *μ*M LA and 100 nM vitD alone or combined reverted previously observed effects indicating the activation of survival pathways. In addition, the combination of LA with vitD showed a greater effect in PI3/Akt activity compared to ERK/MAPK, supporting the hypothesis that after the activation of Akt, all neuronal survival signaling was switched on.

### 3.5. Evaluation of LA and VitD Activities under Iron Accumulation

**Figure 7:**
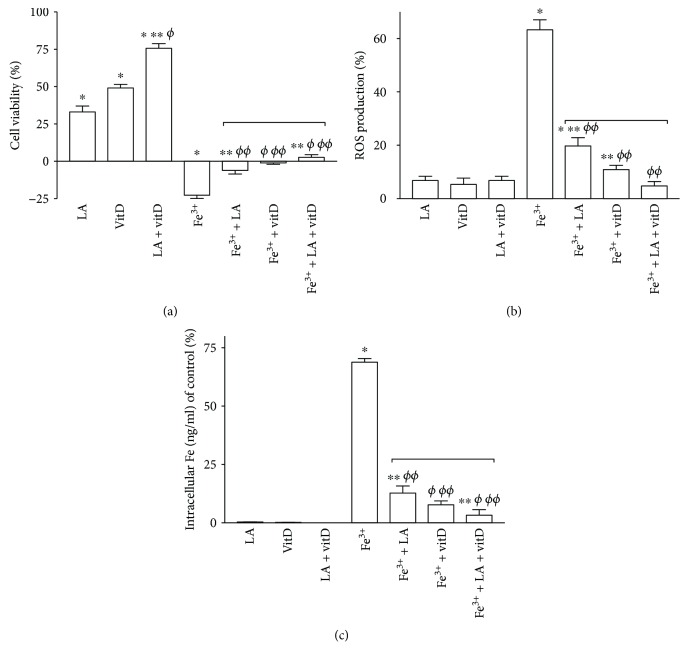
Cell viability, ROS production, and iron quantification in astrocytes: (a) cell viability, (b) ROS production, and (c) the intracellular iron quantification measured on astrocytes pretreated with Fe^3+^ for 6 days and then with vitD and LA alone or combined for additional 6 days. The abbreviations are the same as reported in Figure 1. Data are expressed as means ± SD (%) of five independent experiments normalized to control values (0% line). ^∗^*p* < 0.05 vs. control; ^∗∗^*p* < 0.05 vs. LA; ^*ϕ*^*p* < 0.05 vs. vitD; ^*ϕϕ*^*p* < 0.05 vs. Fe^3+^, bars: *p* < 0.05 between treatments.

Iron can progressively be accumulated into the brain during normal ageing; in neurodegenerative disorders, it can be stored in abnormal accumulations. Since LA is a potent chelator of divalent metal ions and vitD is able to prevent the damage induced by the accumulation, additional experiments were performed on astrocytes to verify their protective ability in the brain. For this reason, cell viability and ROS production were evaluated in astrocytes after 6 days of stimulation, as reported in Figures 7(a) and 7(b). Exposure to 300 *μ*M Fe^3+^ significantly reduced cell viability of about 23% compared to the control (*p* < 0.05); conversely, following posttreatment for additional 6 days with 50 *μ*M LA and 100 nM vitD alone, cell viability leads to control values, confirming their ability to chelate iron and repair the damage (*p* > 0.05). In addition, the concomitant stimulation with LA and vitD was able to amplify the beneficial effect exerted by the single administration (*p* < 0.05), supporting the idea of its possible helpful use during brain damage. Additional experiments on ROS production were performed to investigate the role of oxidation in the presence of iron accumulation. Exposure of astrocytes to 300 *μ*M Fe^3+^ for 6 days significantly increased the intracellular ROS production, as illustrated in Figure 7(b) of about 60% compared to the control (*p* < 0.05). Moreover, posttreatment for additional 6 days with 50 *μ*M LA and 100 nM vitD alone significantly reduced ROS production (about 68% and 83%, respectively, vs. Fe^3+^alone), and the concomitant administration of LA and vitD improved the reduction of the ROS level compared to 300 *μ*M Fe^3+^ of about 92%. These findings indicate the ability of the combination to counteract the oxidative condition caused by iron accumulation. Since the iron accumulation is an important cause of brain damage, it is essential to discover strategies to prevent it; thus, some experiments were carried out to assess the amount of iron which remains inside the cells. As reported in Figure 7(c), 300 *μ*M Fe^3+^ increased the intracellular accumulation after 6 days of stimulation of about 69% compared to the control (*p* < 0.05), indicating that 6 days with an excess of iron was a sufficient condition to create damage in astrocytes. The poststimulation for additional 6 days with 50 *μ*M LA and 100 nM vitD alone significantly reduced iron accumulation (about 81% and 88%, respectively, vs. Fe^3+^alone), and the concomitant administration of LA and vitD improved the prevention of accumulation compared to 300 *μ*M Fe^3+^ of about 95% and compared to the single administration (*p* < 0.05). These findings indicate the ability of the combination to counteract the iron-dependent damage, preventing its accumulation.

**Figure 8:**
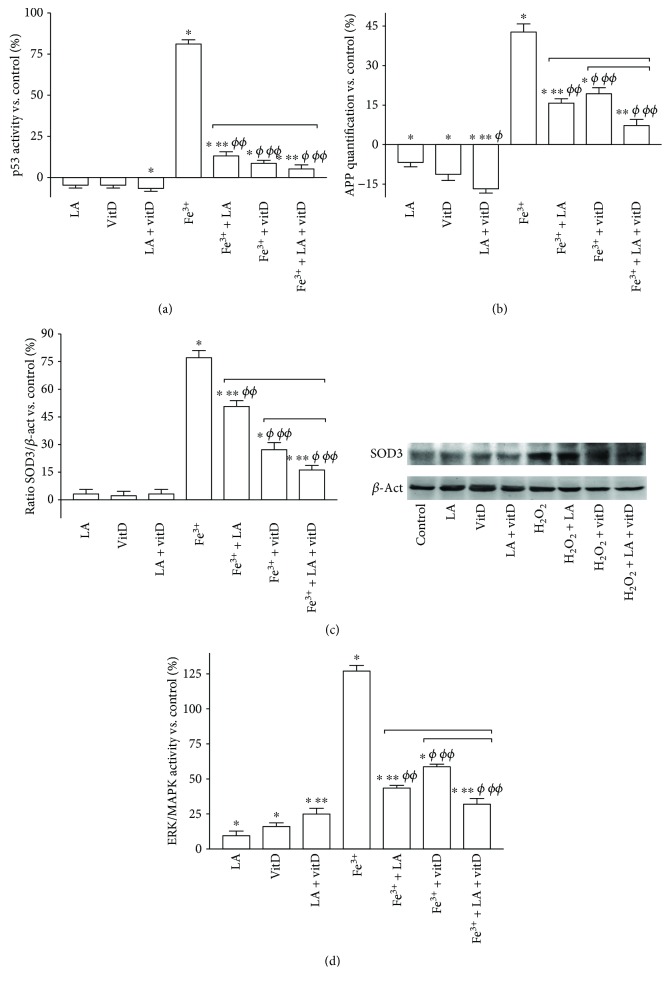
Analysis of the main intracellular pathways analyzed by Western blot and the ELISA kit. In (a), (b), and (d), the measurements of p53, APP, and ERK/MAPK activities, respectively, are reported by the ELISA test. Data are expressed as means ± SD (%) of five independent experiments normalized to control values (0% line). (c) Densitometric analysis and Western blot of cytochrome SOD3 expression obtained in whole astrocyte lysates. Data are expressed as means ± SD (%) of five independent experiments normalized and verified on *β*-actin detection. The abbreviations are the same as reported in Figure 1. ^∗^*p* < 0.05 vs. control; ^∗∗^*p* < 0.05 vs. LA; ^*ϕ*^*p* < 0.05 vs. vitD; ^*ϕϕ*^*p* < 0.05 vs. Fe^3+^, bars: *p* < 0.05 between treatments.

Moreover, to confirm the protection exerted by LA and vitD, p53 activity was analyzed as well. As shown in Figure 8(a), 300 *μ*M Fe^3+^ administration for 6 days caused a significant increase in p53 activity (*p* < 0.05) compared to the control, supporting previous observations about the viability of cells. The poststimulation with 50 *μ*M LA and 100 nM vitD alone for additional 6 days was able to reduce the p53 activation (*p* < 0.05 vs. control) of about 84% and 88%, respectively, compared to 300 *μ*M Fe^3+^. Finally, the combined effect of LA with vitD for additional 6 days after 300 *μ*M Fe^3+^ was able to amplify the reduction on p53 activity (*p* < 0.05) compared to 300 *μ*M Fe^3+^ (increased about 93%) and to the single administration (*p* < 0.05), supporting the beneficial effects previously observed. The production, accumulation, and aggregation of APP during neurodegeneration are influenced by a number of modulators, and among these is iron, so the level of APP under these conditions was also detected. As illustrated in Figure 8(b), exposing astrocytes to 300 *μ*M Fe^3+^ for 6 days significantly increased the APP level of about 43% compared to the control (*p* < 0.05); posttreatment for additional 6 days with 50 *μ*M LA and 100 nM vitD significantly reduced the APP level (about 63% and 55%, respectively, vs. 300 *μ*M Fe^3+^ alone), and the concomitant administration of LA and vitD amplified the reduction compared to 300 *μ*M Fe^3+^, 50 *μ*M LA, and 100 nM vitD alone (*p* < 0.05, about 82%, 53%, and 61%, respectively). These findings confirm the importance of the combination to prevent the iron-dependent damage in the brain. Cellular oxidative stress and antioxidant enzyme dysregulation are linked to age-related brain degeneration, so SOD3 expression was investigated in the same conditions previously reported. The stimulation for 6 days with 300 *μ*M Fe^3+^ caused a significant increase in SOD3 expression (*p* < 0.05) compared to the control which was reduced by the successive stimulation for 6 days more with 50 *μ*M LA or 100 nM vitD alone (*p* < 0.05), indicating a positive effect of 50 *μ*M LA and 100 nM vitD to maintain a correct balance of oxidant activity in astrocytes (Figure 8(c)). In addition, the combined effect of LA with vitD added for 6 days after 300 *μ*M Fe^3+^ was able to significantly reduce the expression of SOD3 (*p* < 0.05) compared to both 300 *μ*M Fe^3+^(about 78%) and 50 *μ*M LA and 100 nM vitD alone (about 67% and 40%, respectively). These data allow to exclude the presence of oxidative damage and inflammatory cascade activation in the presence of iron accumulation, supporting the hypothesis that this combination could be used in human ageing. Finally, the ERK/MAPK activity was investigated in order to demonstrate that the combined stimulation with LA and vitD could prevent iron-dependent cell loss and damage, activating a rescue mechanism such as the survival pathways. Exposing astrocytes to 300 *μ*M Fe^3+^ for 6 days upregulated the ERK/MAPK activation as reported in Figure 8(d) (*p* < 0.05 vs. control), indicating a negative effect and suggesting that iron-dependent ROS generation activates this pathway; poststimulation for additional 6 days with 50 *μ*M LA and 100 nM vitD alone showed a significant decrease compared to 300 *μ*M Fe^3+^ (*p* < 0.05), supporting the beneficial effects observed previously. In addition, the concomitant administration of LA and vitD made the observed attenuation of overexpression more pronounced compared to 300 *μ*M Fe^3+^ (*p* < 0.05), confirming observation of a better survival of astrocytes after the damage.

## 4. Discussion

An ageing brain is characterized by a progressive decline of physiological functions mainly due to the increased imbalance of the antioxidant defense system along with a concomitant increase of oxidative stress [14]. Although ageing cannot be prevented, many studies seek for strategies to counteract or slow it down. Indeed, recent studies hypothesize the use of antioxidant molecules combined with symptomatic drugs to reduce oxidative stress, thus improving cognitive functions during ageing and in age-related diseases [14]. A large body of experimental research indicates that the brain is highly susceptible to oxidative damage due to high concentrations of polyunsaturated fatty acids and transition metals that are involved in the generation of the hydroxyl radical [51, 52]. In an adult brain, astrocytes are responsible in maintaining neuronal and synaptic functions [10]. Oxidative stress plays a key role in astrocyte loss, mainly due to the highly active mitochondria metabolism [14].

According to literature, the brain has a poor catalytic activity and has low levels of protective antioxidant enzymes; for this reason, in this work, the efficacy of LA and vitD in oxidative mechanisms involved in the ageing process was tested. Indeed, it was demonstrated that the combination of LA and vitD exerts a synergistic and cooperative effect on astrocyte activity indicating a possible new strategy to slow down ageing. The combination of LA and vitD is able to perform beneficial effects directly on viability of astrocytes, since these substances are able to cross the blood-brain barrier. Moreover, the combination of LA and vitD increases the absorption rate of the two substances compared to the control during time and to their single administration starting from 60 min, supporting the cooperative effects of LA and vitD also during permeability. A recent work by Farr et al. [29] demonstrated that lipoic acid improves memory and reverses indices of oxidative stress in old mice but decreases the lifespan. This feature has not been studied in this research. This could be a limitation of this research, which could be addressed in the future. However, vitD, with its antioxidant action, allows to use lower doses of lipoic acid, and this could improve the safety of the molecule. Another limitation of this study could be the use of HUVEC rather than a brain-derived endothelial cell. However, some studies indicate that this experimental model can present structural and functional equivalency with in vivo BBB while still using HUVEC [53].

Since the main theory at the basis of brain ageing regards the oxidative stress condition, additional experiments on ROS production were performed. Under oxidative conditions, treatment with LA and vitD improves the reduction of the ROS level. The alteration of the formation of a proton gradient across the inner mitochondrial membrane is considered to be one of the key indicators of cellular viability; the combination of LA and vitD suppressed the effect of H_2_O_2_-induced mitochondrial dissipation. Thus, this study demonstrates that combined LA and vitD can attenuate the H_2_O_2_-induced apoptosis through the mitochondrial-mediated pathways. Furthermore, the specifics to be used in combination have been determined evaluating the intracellular pathways activated by LA and vitD during oxidative condition. Our findings suggest that there is a reduction on p53 activity after the stimulation with LA and vitD that it is correlated to survival-favored conditions. Moreover, the involvement of the caspase pathway has been examined analyzing the expression of cytochrome C. The combination of LA and vitD is able to reduce cytochrome C expression during oxidative damage, supporting mitochondrial integrity. Besides, it has been demonstrated that stimulation with LA and vitD under oxidative condition can reduce cell death and increase cell survival, by the activation of ERK and Akt mediators. Even the major pathways (SOD3 and iNOS) involved in oxidative stress were inhibited by administration of LA and vitD, indicating better cell survival.

Since neuroinflammation is a common cause of brain ageing, PKA, a key anti-inflammatory marker, was also investigated under the same conditions. The cAMP-dependent PKA signaling is one of the best-characterized signal transduction mechanisms in the central nervous system [54] and plays a diverse role in neuronal functions, such as regulating synaptic plasticity, learning, and memory [55]. PKA is a key phosphorylating enzyme, which triggers a wide variety of physiological involvement in cell survival, synaptic plasticity, and activation or repression of gene expression [54]. The PKA role in inflammation consists in its involvement in the cross talk between different signaling mechanisms such as the inactivation of phospholipase C*β*; the phosphorylation of inositol 1,4,5-tris phosphate receptors, thereby modulating Ca^2+^ influx; and the phosphorylation of calcium-calmodulin kinase. In addition, cAMP stimulates mitogen-activated protein kinase activity in cultured neurons [54]. In addition, some studies by Salinthone et al. demonstrated that cAMP/PKA inhibits NF-*κ*B function by slowing down its translocation into the nucleus and inhibits NF-*κ*B activity [56].

The expression of PKA observed in astrocytes showed a significant increase in the presence of LA and vitD, and their combination amplified this effect, supporting the hypothesis of an anti-inflammatory activity of combined LA and vitD. The combination of LA and vitD added after H_2_O_2_ had a similar effect on PKA expression. This finding allows to exclude the involvement of inflammation cascade during treatment with LA and vitD combined.

Neuroinflammation and iron accumulation are hallmarks of a variety of adult neurodegenerative diseases [57], and particularly, iron is recognized to influence the biochemistry of proteins involved in neurodegeneration (for instance, APP), as well as those playing a crucial role in neuronal development and efficiency [58]. Moreover, it was demonstrated that the exposure to Fe^3+^ significantly reduced cell viability and increased ROS production and the combination of LA and vitD can prevent Fe^3+^-dependent oxidative injury in astrocytes. It is well known that LA is a potent chelator of divalent metal ions and vitD is able to prevent the damage induced by the iron accumulation [41, 59]. However, processes involved in age-related and disease-related accumulations of iron and iron-induced inflammation in specific brain regions and cells are poorly understood. Posttreatment with LA and vitD was able to restore the damage caused by iron accumulation. Furthermore, it has been demonstrated that LA and vitD alone or together were able to prevent intracellular iron accumulation damage by their ability to prevent iron deposition promoting its elimination (in fact, the treatment was added after 6 days of iron administration). Besides, even if the presence of iron caused a harmful condition for the cells, the response to treatment significantly activated the cellular survival mechanisms (ERK) by switching off apoptosis mechanisms (p53). Indeed, these effects were found to be mediated by the inhibiting effect of LA on oxidative (SOD3) and inflammatory (APP) systems.

## 5. Conclusions

In conclusion, this study demonstrates for the first time that the combination of LA and vitD is an effective treatment for astrocytes under oxidative stress conditions, indicating the possibility of developing new strategies to treat brain ageing in all stages. Besides, the combined treatment with LA and vitD improved the negative effects of preneurodegenerative conditions, so there are the preludes to develop a new formulation to slow down brain ageing and neurodegenerative diseases, like AD and PD, such as a new food supplement Cebral®.

## Data Availability

All data reported have been obtained from experiments carried out in the authors' laboratory.
